# Antibiogram profile of *Enterococcus faecalis* and *Enterococcus faecium* in chicken meat from supermarkets in Sleman District, Indonesia

**DOI:** 10.14202/vetworld.2025.491-499

**Published:** 2025-02-27

**Authors:** Andi Muhamad Isra Nurrahmat, Heru Susetya, Khrisdiana Putri

**Affiliations:** 1 Veterinary Science Postgraduate Programme, Faculty of Veterinary Medicine, Universitas Gadjah Mada, Indonesia; 2 Department of Veterinary Public Health, Faculty of Veterinary Medicine, Universitas Gadjah Mada, Indonesia

**Keywords:** antibiotic resistance, *Enterococcus*, food safety, multidrug resistance, supermarket chicken

## Abstract

**Background and Aim:**

Enterococci are commensal bacteria in the digestive tract of poultry and serve as indicators of fecal contamination. Their significance in veterinary and human medicine arises from their ability to acquire antibiotic-resistance genes, posing a potential public health risk. Poultry meat, a major protein source in Indonesia, can act as a reservoir for *Enterococcus* species, transferring antibiotic-resistant strains to humans through food handling. Despite rigorous hygiene standards in supermarket supply chains, limited studies have assessed contamination levels. This study aimed to identify *Enterococcus* species from supermarket chicken meat in Sleman District, Yogyakarta, Indonesia, and evaluate their antibiotic resistance profiles.

**Materials and Methods:**

Chicken breast samples were randomly collected from three Supermarkets (A, B, and C). Bacterial isolation was performed using buffered peptone water and enterococcosel agar. Presumptive colonies were confirmed by polymerase chain reaction for genus and species identification. Antibiotic susceptibility was assessed using the Kirby–Bauer disk diffusion method against ampicillin (AMP), tetracycline (TET), erythromycin (ERY), and vancomycin (VAN).

**Results:**

A total of 269 *Enterococcus* isolates were confirmed, including 163 *Enterococcus faecium* (EFM), 92 *Enterococcus faecalis* (EFS), and 14 other *Enterococcus* species. Resistance to AMP, TET, and ERY in EFM was 12.12%, 57.57%, and 66.67%, respectively, while resistance in EFS was 4.54%, 31.82%, and 63.63%. No isolates showed resistance to VAN. Multidrug resistance (MDR) was observed in 60.60% of EFM and 36.36% of EFS isolates.

**Conclusion:**

Despite high susceptibility to AMP and VAN, resistance to TET and ERY was prevalent. The presence of MDR isolates underscores the need for continuous surveillance of antibiotic resistance in *Enterococcus* species within the food chain. This study highlights the necessity of further research with expanded sampling and antibiotic panels to assess the dissemination of antibiotic resistance genes and potential public health risks.

## INTRODUCTION

Enterococci are Gram-positive bacteria commonly found in chicken meat [1–3]. These bacteria are commensals in the digestive tract of various animals, including chickens [4], and they can contaminate chicken meat during slaughter and unhygienic meat handling processes [1]. Enterococci are also known as the main agents of nosocomial infections, and *Enterococcus faecalis* (EFS) and *Enterococcus faecium* (EFM) are the most isolated species from human patients [5]. Although there is no proof that chicken enterococci has caused existing clinical cases, Manson *et al*. [6] reported that some resistant isolates of chicken-meat enterococci were closely related to clinical isolates and shared virulence factors.

The emergence of antibiotic-resistant bacteria is influenced by antibiotic misuse practices in the poultry rearing industry [7] for prophylaxis and growth promotion [8]. Some countries have been banning antibiotics as growth promoters (AGP) [9], including Indonesia, since early 2018 under the decree of the Ministry of Agriculture of Indonesia no. 14/PERMENTAN/PK.350/5/2017. Before the law, AGP was a common practice. Surveys on broiler farms in West Java, East Java, and South Sulawesi revealed that approximately 80% of farmers were administering antibiotics for non-treatment purposes without a veterinarian prescription [10–12]. Chicken-derived *Enterococcus* can act as a vehicle for transferring antibiotic-resistance genes to other bacteria [2, 13]. However, the US, Canada, Korea, Bangladesh, and Turkey have reported high enterococcal contamination of supermarket chicken meat [13–17]. These findings further highlight the potential risk of the spread of antibiotic resistance genes to humans.

Consumers in Indonesia have rights to safety and convenience in consuming food, and this right is regulated in Law No. 8 of 1999 on consumer protection. As the final point of contact between the food supply chain and consumers, retail outlets play a crucial role in ensuring the distribution of safe food [18]. The safety of food of animal origin sold in retail establishments is intended to be guaranteed by the Ministry of Agriculture Regulation (Permentan) 11/2020, which mandates the issuance of a Veterinary Control Number (Nomor Kontrol Veteriner [NKV]) to eligible business units. The possession of an NKV by a retail outlet or supermarket signifies that the NKV has met the hygiene and sanitation requirements established for the safe handling of animal products within its business operations.

No available studies have reported the antibiogram profile of enterococci isolated from supermarket chicken-meat in Yogyakarta, Indonesia. Published studies on antibiotic resistance bacteria isolated from supermarket chicken meat in Indonesia are limited to *Staphylococcus aureus, Salmonella* spp*., Escherichia coli*, and *Campylobacter* [17, 19–23]. Although enterococci may not be primarily recognized as outbreak pathogens, they play a significant role in the spread of antibiotic-resistant genes [4]. This study aimed to identify and characterize *E. faecalis* and *E. faecium* isolates from supermarket chicken meat in Sleman District, Yogyakarta, Indonesia, and to evaluate their antibiotic resistance profiles, with a particular focus on Multidrug resistance (MDR) patterns and potential public health implications.

## MATERIALS AND METHODS

### Ethical approval

This study qualified for ethical exemption as data collection, excluding laboratory testing, was exclusively observational and involved no live animal or human subjects. The chicken meat samples were purchased from supermarkets.

### Study period and location

This study was conducted from March to December 2023 at the Veterinary Public Health Laboratory, Faculty of Veterinary Medicine, Universitas Gadjah Mada, Yogyakarta, Indonesia.

### Sample collection

Chicken breast meat samples were randomly purchased from 3 Supermarkets (A, B, and C) located in the Sleman district, Yogyakarta. Twenty samples were collected from Supermarket A (Mlati sub-district), 20 samples were collected from Supermarket B (Depok sub-district), and eight samples from Supermarket C (Depok sub-district). Samples were kept aseptically at cold temperatures during transportation to the Veterinary Public Health laboratory of the Faculty of Veterinary Medicine, Universitas Gadjah Mada Yogyakarta for testing.

### Bacterial isolation

The bacterial isolation was conducted as described by Aslam *et al*. [13] with some modifications. Enrichment in enterococcosel broth was omitted in this study; after overnight incubation, the suspension was directly streaked onto Enterococcosel agar. Suspected *Enterococcus* colonies were directly subjected to PCR for genus and species identification. A 25-g chicken meat sample was suspended in 225 mL of buffered peptone water (Merck, Germany) and incubated for 18 h at 42°C. Bacterial growth was indicated by the turbidity of the media. The suspension was then streaked onto Enterococcosel agar (BBL Becton, France) and incubated at 37°C for 24 h. A total of 6 presumptive *Enterococcus* colonies were then subcultured onto another Enterococcosel agar plate (BBL Becton) and incubated at 37°C for 24 h. The selected presumptive colonies were subsequently grown in brain-heart-infused broth (Oxoid, UK) frozen with 50% glycerol to serve as stock and molecular templates.

### Molecular identification of *Enterococcal* species

Molecular screening for *Enterococcus* genus and species was conducted using polymerase chain reaction (PCR) and the primers listed in Table 1 [24–26]. The genus was determined using the *tuf gene* (112 bp), as described by Mwikuma *et al*. [24]. The cycling condition was initialized with denaturation at 95°C for 3 min, followed by 35 cycles of denaturation at 95°C for 30 s, annealing at 55°C for 30 s, and elongation at 72°C for 1 min. The final extension step was performed at 72°C for 7 min. Samples tested positive for the genus were screened for EFS, EFM, and ECO. The EFS and EFM were identified by PCR amplification of the *ddl* gene, as described by Arabestani *et al*. [25]. The target amplicon sizes for EFS and EFM were 941 bp and 658 bp, respectively. PCR was initiated with denaturation at 94°C for 5 min, followed by 35 cycles of denaturation at 94°C for 1 min, annealing at 54°C for 1 min, elongation at 72°C for 1 min, and finalization with an extension at 72°C for 10 min. The identification of *Enterococcus cecorum* (ECO) was performed by targeting the 371 bp *sodA* gene, as described by Dolka *et al*. [26]. The amplification cycle for ECO started with denaturation at 95°C for 4 min, followed by 35 cycles of denaturation at 95°C for 30 s, annealing at 54°C for 1 min, elongation at 72°C for 1 min, and ending with a final extension at 72°C for 7 min. The PCR mixture (GoTaq(R) Green, USA) of 25 μL total volume consisted of 12.5 μL 2X GoTaq® Green Master Mix, 1 μL of each primer (40 μM), 9.5 μL nuclease-free water, and 1 μL of bacteria glycerol stock. Amplicons were visualized on 2% agarose gels (Genetics Science, 1^st^ BASE, Singapore).

**Table 1:** Primers used in the polymerase chain reaction test.

Primer	Sekuens (5’ – 3’)	Size (bp)	Position	Reference
Genus (*tuf*)	F TAC TGA CAA ACC ATT CAT GAT G R AAC TTC GTC ACC AAC GCG AAC	112	618–639	[24]
*Enterococcus faecalis* (*ddl*)	F ATC AAG TAC AGT TAG TCT R ACG ATT CAA AGC TAA CTG	941	708–729	[25]
*Enterococcus faecium* (*ddl*)	F TTG AGG CAG ACC AGA TTG ACG R TAT GAC AGC GAC TCC GAT TCC	658	98–116	[25]
*Enterococcus cecorum* (*sodA*)	F AAA CAT CAT AAA ACC TAT TTA R AAT GGT GAA TCT TGG TTC GCA	371	1038–1021	[26]

### Antibiotic sensitivity test (AST)

The test was conducted according to the Clinical and Laboratory Standards Institute (CLSI) [27]. The antibiotics used were vancomycin (VAN) 30 μg (van30), erythromycin (ERY) 15 μg (ery15), tetracycline (TET) 30 μg (tet30), and ampicillin (AMP) 10 μg (amp10). These antibiotic panels are widely used in both human and veterinary medicine [2, 13, 14, 28].

The overnight *Enterococcal* isolate in BPW was adjusted to 0.5 McFarland turbidity and evenly spread onto Mueller–Hinton Agar (Oxoid, United Kingdom). Subsequently, antibiotic disks were placed on the surface of the inoculated agar and incubated for 24 h at 37°C. The results were interpreted based on the size of the inhibition zone formed around the disk, as described by CLSI [27].

### Statistical analysis

All data were analyzed using GraphPad Prism version 10.1.1 for Macintosh (GraphPad Software, Boston, Massachusetts USA, www.graphpad.com). Descriptive statistics were used to summarize the prevalence of EFM and EFS, as well as their antibiotic resistance patterns.

The Fisher’s exact test was applied to assess significant differences in antibiotic resistance rates between EFM and EFS isolates. The prevalence of MDR was compared across supermarkets using a one-way analysis of variance. Normality was checked using the Shapiro–Wilk test.

For pairwise comparisons of resistance rates among supermarkets, *post hoc* analysis (Bonferroni test) was conducted if significant differences were found. A logistic regression model was used to explore associations between antibiotic resistance and supermarket location.

All statistical tests were two-tailed, with a significance threshold set at p < 0.05. Data are presented as mean ± standard deviation or median (interquartile range) when appropriate.

## RESULTS

### Isolation and identification of *Enterococcus*

**Figure 1:**
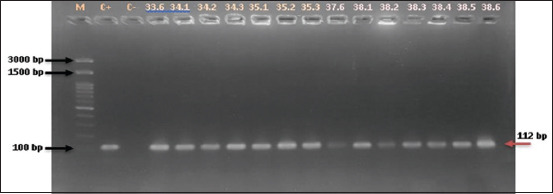
*Enterococcus* genus (112 bp) determination in polymerase chain reaction using bacterial glycerol stock as a template. M=Marker, C+=Positive control, C−=Negative control, lanes afterward are tested isolates.

**Figure 2:**
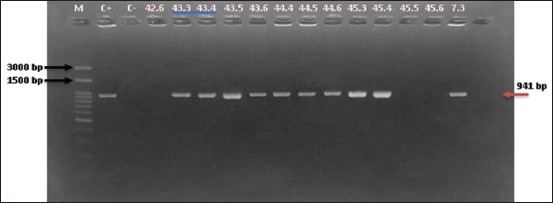
EFS species identification based on *ddl* gene (941 bp). M=Marker, C+=Positive control, C−=Negative control, 42.6, 45.5, and 45.6 are tested negative for EFS; remaining lanes are tested positive for EFS. EFS=*Enterococcus faecalis*.

**Figure 3:**
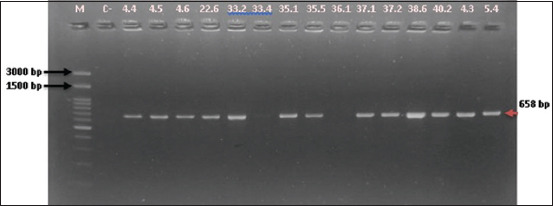
EFM species identification based on *ddl* gene (658 bp). M=Marker, C−=Negative control; 33.4, 36.1, are tested negative for EFM; remaining lanes are tested positive for EFM. EFM*=Enterococcus faecium*.

**Figure 4:**
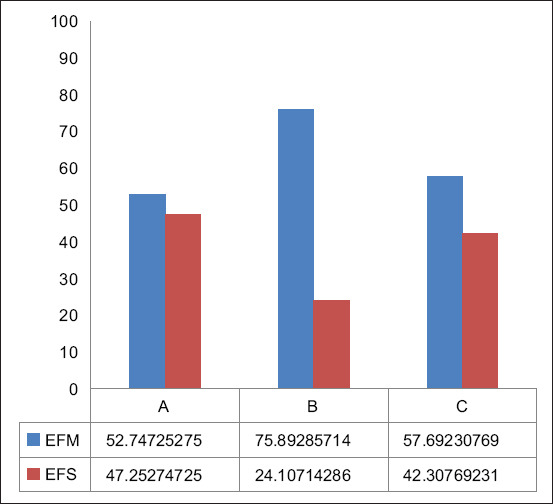
*Enterococcus* species distribution across supermarket collections. Supermarkets A and C exhibited similar proportions of EFM and EFS. In contrast, Super market B showed a three-fold higher percentage of EFM than EFS. EFM*=Enterococcus faecium*, EFS=*Enterococcus faecalis*.

A total of 271 presumptive *Enterococcus* colonies were selected for further analysis. Small, transparent colonies with zones ranging from brownish black to black are the hallmarks of *Enterococcus* colonies on Enterococcosel agar (BBL Becton). The number of genus-confirmed colonies by PCR was 269/271 (99.26%) colonies. Of the 269 colonies, 163 (60.59%) were identified as EFM, 92 (34.20%) as EFS, and 14 (5.20%) were unable to be detected as either EFM, EFS, or ECO (Table 2). The visualization of the amplification results for the detected genera and species are shown in Figures 1–3. The highest proportion of EFM (75.89%) was identified in isolates collected from Supermarket B, whereas EFS was identified from Supermarket A (47.25%) (Figure 4). Of the 48 chicken meat samples analyzed, EFS and EFM were detected in 26 samples. In the remaining samples, only EFS or EFM was detected. There was a statistically significant association between the type of *Enterococcus* strain and the supermarket (p = 0.0015). Analysis of MDR prevalence across supermarkets revealed contrasting results for EFS and EFM. While EFS showed no significant difference in prevalence (p = 0.8406), EFM exhibited a significant variation (p = 0.0184). Drug resistance patterns in EFM and EFS, considering combinations of one, two, and three drugs, did not vary significantly among supermarkets (p = 0.2800). Similarly, supermarket location did not significantly predict MDR in these bacteria, although a trend toward association was observed (p = 0.0705).

**Table 2:** The proportion of *Enterococcal* genera and species identified by polymerase chain reaction.

Genus	Number of isolates	*Enterococcus faecalis*	*Enterococcus faecium*	*Enterococcus cecorum*	Other *Enterococcus*
*Enterococcus* (%)	269 (99.26)	92 (34.20)	163 (60.59)	0	14 (5.20)
Non-enterococcus (%)	2 (0.74)				
Total isolate	271				

### AST

**Figure 5:**
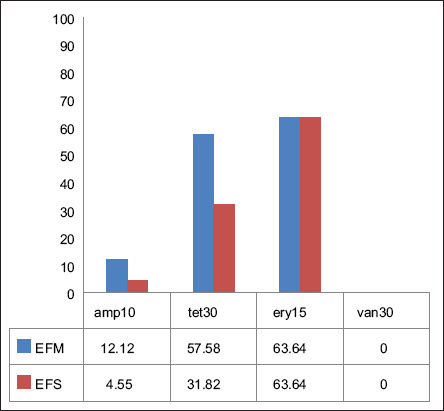
Antibiotic resistance in supermarket-chicken meat *Enterococcus* species. The EFM and EFS groups exhibited similar resistance rates to ERY. Notably, both species were susceptible to vancomycin. EFM*=Enterococcus faecium*, EFS=*Enterococcus faecalis*, ERY*=*Erythromycin.

The sensitivity test to amp10, ery15, tet30, and van30 was performed on 55 isolates, comprising 33 EFM and 22 EFS isolates from all supermarkets. The test was performed on both EFM and EFS isolates when both species were recovered from the same sample. However, if only one species (either EFM or EFS) was isolated from a sample, only that isolate was subjected to AST. A statistically significant difference (p = 0.007) was observed in resistance rates to AMP10, ERY15, TET30, and VAN30 among the supermarkets in EFM and EFS. A high proportion of EFM and EFS isolates remained susceptible to amp10 (87.88% and 95.45%, respectively). However, resistance to tet30 was more prevalent, with only 36.36% of EFM isolates and 50% of EFS isolates remaining sensitive. Resistance to ery15 was also observed, with only 18.18% of EFM isolates and 22.73% of EFS isolates showing sensitivity. Notably, all isolates remained susceptible to van30 (Figure 5 and Table 3).

**Table 3:** Antibiotic sensitivity tests on *Enterococcus* isolates representing all supermarkets.

Antibiotics	*Enterococcus faecium* (n = 33) (%)			*Enterococcus faecalis* (n = 22) (%)		
	
	S*	I*	R*	S*	I*	R*
Ampicillin 10	87.88 (29/33)	0	12.12 (4/33)	95.45 (21/22)	0	4.55 (1/21)
Tetracycline 30	36.36 (12/33)	6.06 (2/33)	57.58 (19/33)	50.00 (11/22)	18.18 (4/22)	31.82 (7/22)
Erythromycin 15	18.18 (6/33)	18.18 (6/33)	63.64 (21/33)	22.73 (5/22)	13.64 (3/22)	63.64 (14/22)
Vancomycin 30	100 (33/33)	0	0	100 (22/22)	0	0

* S=Sensitive, I=Intermediate, and R=Resistant

A total of 37 (67.27%) isolates manifested resistance to at least one antibiotic (Table 4). Both EFM and EFS demonstrated equal percentages of MDR by 66.67% and 63.64% for isolates resistant to multiple antibiotics. There was no significant difference (p = 0.1024) pattern between EFM and EFS in resistance manifestation. A particularly high distribution of MDR isolates was observed in Supermarket C (85.71%), followed by Supermarket B (68.75%) and Supermarket A (33.34%).

**Table 4:** The proportion of EFM and EFS resistant to at least one antibiotic.

Resistance percentage	EFM (n = 33)	EFS (n = 22)
Resistance to 1 antibiotic	3 (12.12)	6 (27.27)
Resistance to 2 antibiotics	17 (51.51)	8 (36.36)
Resistance to 3 antibiotics	2 (6.06)	0
Total	22	14

EFM=*Enterococcus faecium*, EFS=*Enterococcus faecalis*

## DISCUSSION

### Chicken-meat *Enterococcus* molecular identification and proportion

EFM and EFS are the most common species found in chicken meat [1]. This study described the antibiogram profile of chicken-derived *Enterococcus* isolated from premium retail businesses in Indonesia. Furthermore, this study demonstrated robust molecular detection for the genus and species directly from bacteria glycerol stock, eliminating the need for DNA extraction. This method also avoids the reliance on costly and laborious biochemical tests, making it valuable for high-throughput screening of numerous samples.

The proportion of *Enterococcus* species identified in this study is consistent with previous findings; Manson *et al*. [6], Parmar *et al*. [29], and Tyson [30] reported 98.23%, 95.23%, and 95%, prevalence of *Enterococcus* isolated from chicken meat, respectively. However, other researchers have reported lower percentages, as reported by Bin Kim *et al*. [14] (77.7%), Samad *et al*. [16] (35%), Martinez-Laorden *et al*. [31] (66%), and Onaran *et al*. [32] (30%). Furthermore, the proportion of EFM in this study was higher than that of EFS. These findings are consistent with those of Sanlibaba *et al*. [12] and Tyson [30]. Nevertheless, Miranda *et al*. [3], Manson *et al*. [6], and Kim and Ahn [33] reported a higher percentage of EFS than EFM. The variation in predominant *Enterococcus* species may be influenced by the use of antibiotics in poultry-rearing systems. Miranda *et al*. [3] observed a higher proportion of EFS (36.67%) in organic compared to conventional (21.67%) systems, while the proportion of EFM was comparable between the two systems. Similarly, Bin Kim *et al*. [14] and Kim *et al*. [33] reported a higher percentage of EFS in organic chicken (76.9%) than in conventional (57.7%). Conversely, EFM was more prevalent in conventional (11.5%) than organic chicken (3.8%) systems.

ECO was not detected in this study despite being a common commensal bacterium in the chicken digestive tract alongside EFS and EFM. Previous studies have consistently shown that the prevalence of ECO and other *Enterococcus* species in chicken meat is lower compared to EFS and EFM. This observation may be attributed to the findings of Lebreton *et al*. [7], who demonstrated that EFS and EFM exhibit greater resistance to desiccation and starvation compared to other *Enterococcus* species. A total of 14 isolates in this study were confirmed as *Enterococcus* isolates but could not be identified as EFM, EFS, or ECO. These isolates may reside in other *Enterococcus* species commonly found in chicken meat, such as *Enterococcus hirae, Enterococcus durans, Enterococcus gallinarum, Enterococcus avium*, and *Enterococcus casseliflavus* [2, 34].

Although EFS and EFM are typical species found in the digestive tracts of animals and are frequently isolated from meat, Torres *et al*. [40] have described animal infections associated with these species. To date, these species have been primarily recognized as human pathogens. Several studies by Aslam *et al*. [13], Hammerum [35], Torres *et al*. [40], and Harada *et al*. [43] have investigated the potential for the transmission of antimicrobial-resistant Enterococci between animals and humans. Manson *et al*. [6] have characterized chicken meat-derived enterococci that exhibited high genetic similarity to clinical strains. These findings suggest that chicken-meat-associated strains can cause clinical infections in humans through direct transmission.

### Antibiotic resistance profiling of *Enterococcus*

The emergence of resistant isolates from food animals and their products is strongly linked to the misuse of antibiotics in animal-rearing practices [33]. The use of antibiotics for non-treatment purposes, such as growth promoters, was a prevailing practice in Indonesian broiler farms [11, 36].

Most isolates were resistant to ERY and TET. Several studies of EFS and EFM from chicken meat showed high resistance to ERY and TET in addition to lincomycin, kanamycin, streptomycin, and rifampin [6, 14, 28]. ERY and TETs, including doxycycline, oxytetracycline, and chlortetracycline, are generally used in the poultry industry [37]. Bin Kim *et al*. [14] and Aslam *et al*. [13] have isolated EFS from chicken meat harboring resistance genes for clinically relevant antibiotics such as macrolides, TETs, streptogramin, bacitracin, and lincosamides. A 2017 survey by the Center for Veterinary Drug and Feed Monitoring reported that the use of ERY and TETs in chicken farms across West Java, Central Java, and East Java reached 37.5%. Furthermore, a 2017 Ministry of Agriculture survey conducted on broiler farms in West Java, East Java, and South Sulawesi revealed that ERY and TETs were among the top 10 most frequently used antibiotics by farmers [36].

In this study, <10% of *Enterococcus* isolates exhibited amp resistance, with a higher proportion observed in EFM. This finding is in contrast with previous studies by Rebelo *et al*. [4] and Kilonzo-Nthenge *et al*. [28], which reported higher AMP resistance rates in chicken-meat-derived EFM (33% and 23.2%, respectively). Among the antibiotics tested in this study, *Enterococcus* spp. demonstrated the lowest resistance to AMP compared with Tet and Ery. This finding suggests the continued effectiveness of AMP against enterococci, which is consistent with Zacharopoulos *et al*. [38].

AMP, a member of the beta-lactam class of antibiotics, maintains susceptibility to many enterococci, whereas most other beta-lactams exhibit intrinsic resistance. AMP resistance is more frequently observed in clinical isolates of EFM than in EFS [39, 40]. In EFM, AMP resistance can arise from increased expression of the low-affinity penicillin-binding protein (PBP5), resulting in weakened binding to beta-lactam antibiotics. Alternatively, mutations within the *pbp5* gene, leading to amino acid substitutions in or near the enzyme’s active site, can also confer resistance [41].

No VAN-resistant isolates were detected in this study. This is consistent with the fact that VAN and other glycopeptide antibiotics are not approved for use in food animals in Indonesia. VAN is primarily used in human medicine to treat Gram-positive bacterial infections, particularly those caused by Enterococci. Although a case of VAN-resistant Enterococci has been reported in an Indonesian patient [42], these findings suggest that the acquisition and spread of VAN resistance determinants may be primarily confined to hospital and healthcare settings in the country. However, Harada *et al*. [43] identified VAN resistance ECO isolated from chicken. A total of 2 (6.06%) EFM isolates originating from Supermarkets B and C exhibited MDR to AMP, TET, and ERY. This finding is consistent with the higher percentage (28%) of MDR EFM reported by Rebelo *et al*. [4]. Kilonzo-Nthenge *et al*. [28] also reported MDR involving TET, ERY, and penicillin. Furthermore, Bin Kim *et al*. [14] identified TET and ERY as antibiotics with the most prevalent MDR characteristics. In addition, their study revealed that >50% of EFS isolates exhibited MDR involving TET and ERY.

The Veterinary Control Number (NKV), issued by the Ministry of Agriculture of Indonesia, guarantees the safety, hygiene, and sanitation of food animal products within a business unit. Although these supermarkets possess NKV certification, the antibiotic-resistant bacteria are still observed. This finding underscores the capacity of Enterococci to persist within the food system and act as carriers for the transfer of resistance genes from the farm ecosystem to the human environment. Personal protective equipment and consistent hygiene practices after handling raw meat are crucial for minimizing the risk of transmitting antibiotic-resistant bacteria to humans. Antibiotic resistance poses a significant threat to public health and requires immediate attention. Several key strategies can be implemented to mitigate the emergence of antibiotic-resistant bacteria in animals and their products. Key strategies to mitigate the emergence of antibiotic-resistant bacteria in animals and their products include robust implementation of biosecurity measures and adhering to good animal husbandry practices; implementing strict regulations on antibiotic sales and distribution; ensuring that antibiotics are only available through valid veterinary prescriptions; and conducting comprehensive educational programs for farmers to raise awareness about the impact of antibiotic misuse on both animal and human health.

## CONCLUSION

This study provides an assessment of the antibiotic resistance profile of *Enterococcus* species isolated from supermarket chicken meat in Sleman District, Yogyakarta, Indonesia. A total of 269 *Enterococcus* isolates were identified, comprising 163 EFM, 92 EFS, and 14 other *Enterococcus* species. Resistance to ERY (66.67% in EFM, 63.64% in EFS) and TET (57.57% in EFM, 31.82% in EFS) was notably high, while VAN resistance was not detected. MDR was observed in 60.60% of EFM isolates and 36.36% of EFS isolates, highlighting the potential role of supermarket chicken meat as a vehicle for antimicrobial resistance transmission.

This study is the first to assess *Enterococcus* contamination in supermarket chicken meat in Yogyakarta, providing baseline data for future research. The robust molecular identification approach using PCR ensured accurate species-level detection, eliminating the need for labor-intensive biochemical tests. The study also employed comprehensive antimicrobial susceptibility testing using key antibiotics commonly utilized in both human and veterinary medicine. The findings emphasize the potential transmission of antibiotic-resistant *Enterococcus* strains from the food chain to humans, reinforcing the public health significance of antimicrobial resistance monitoring.

However, limitations include a relatively small sample size, as only three supermarkets were surveyed, which may not fully represent the overall supermarket chicken meat contamination in Indonesia. The study also assessed a limited antibiotic panel (AMP, TET, ERY, and VAN), whereas a broader spectrum of antibiotics could provide a more detailed resistance profile. In addition, genetic characterization of resistance determinants was not performed, limiting insights into the mechanisms underlying the observed resistance patterns. Furthermore, the study did not investigate potential transmission routes, such as cross-contamination during handling or packaging, which could provide more insight into consumer exposure risks.

Future studies should expand sample collection across multiple retail chains and traditional markets to compare resistance prevalence in different distribution channels. Whole-genome sequencing or PCR-based detection of resistance genes should be incorporated to identify genetic determinants and mechanisms of antibiotic resistance. Investigating farm-to-retail contamination pathways, including biosecurity practices, slaughterhouse hygiene, and storage conditions, could help identify critical control points for limiting bacterial contamination. Longitudinal studies should be conducted to assess resistance trends over time, while public health risk assessments, including comparisons with human clinical isolates, will help evaluate the potential direct impact of meat-associated *Enterococcus* on human infections.

This study underscores the urgent need for continuous surveillance of antibiotic resistance in the food chain and reinforces the One Health approach in tackling antimicrobial resistance at the animal-human-environment interface. Strengthening food safety regulations, improving hygiene practices in the poultry supply chain, and restricting the misuse of antibiotics in animal farming will be critical steps toward mitigating the spread of antibiotic-resistant *Enterococcus* species.

## AUTHORS’ CONTRIBUTIONS

KP and AMIN: Conceived and designed the study. KP: Optimized bacteria isolation and molecular identification. AMIN: Collected samples and isolation and molecular identification. KP and AMIN: Drafted the manuscript. KP, AMIN, and HS: Analyzed and interpreted the data. All authors have read and approved the final manuscript.
